# The Development of Aptamer-Based Gold Nanoparticle Lateral Flow Test Strips for the Detection of SARS-CoV-2 S Proteins on the Surface of Cold-Chain Food Packaging

**DOI:** 10.3390/molecules29081776

**Published:** 2024-04-13

**Authors:** Xiaotong Li, Jiachen Wang, Ge Yang, Xiaona Fang, Lianhui Zhao, Zhaofeng Luo, Yiyang Dong

**Affiliations:** 1 Laboratory of Food Safety and Risk Assessment, College of Life Science and Technology, Beijing University of Chemical Technology, Beijing 100029, China; 2021210678@buct.edu.cn (X.L.); 2021201147@buct.edu.cn (J.W.); 2020400269@buct.edu.cn (L.Z.); 2 CAMS Key Laboratory of Antiviral Drug Research, Beijing Key Laboratory of Antimicrobial Agents, NHC Key Laboratory of Biotechnology of Antibiotics, Institute of Medicinal Biotechnology, Chinese Academy of Medical Sciences and Peking Union Medical College, Beijing 100050, China; yangge@imb.cams.cn; 3 Department of Basic Medicine, Anhui Medical College, Hefei 230601, China; memoryna@mail.ustc.edu.cn; 4 Key Laboratory of Zhejiang Province for Aptamers and Theragnostic, Aptamer Selection Center, Hangzhou Institute of Medicine (HIM), Chinese Academy of Sciences, Hangzhou 310022, China

**Keywords:** SARS-CoV-2, S protein, COVID-19, lateral flow test strips, AuNPs, aptamer

## Abstract

The COVID-19 pandemic over recent years has shown a great need for the rapid, low-cost, and on-site detection of severe acute respiratory syndrome coronavirus 2 (SARS-CoV-2). In this study, an aptamer-based colloidal gold nanoparticle lateral flow test strip was well developed to realize the visual detection of wild-type SARS-CoV-2 spike proteins (SPs) and multiple variants. Under the optimal reaction conditions, a low detection limit of SARS-CoV-2 S proteins of 0.68 nM was acquired, and the actual detection recovery was 83.3% to 108.8% for real-world samples. This suggests a potential tool for the prompt detection of SARS-CoV-2 with good sensitivity and accuracy, and a new method for the development of alternative antibody test strips for the detection of other viral targets.

## 1. Introduction

SARS-CoV-2 is a human-infecting β-coronavirus that was found in Wuhan in December 2019 [1]. The disease caused by its infection was initially named 2019-nCoV by the World Health Organization (WHO) on 12 January 2020, and then formally named COVID-19, or novel coronavirus pneumonia, on 1 February [2]. According to the WHO, more than 770 million cases have been reported, with 7 million reported deaths [3]. In this outbreak, SARS-CoV-2 from an unknown animal source, possibly a seafood market, might have crossed the species barrier to infect humans [4]. Although the likelihood of food-to-human transmission is considered lower than other ways, such as respiratory droplets and cs, it should not be neglected as a risk factor given the large volumes of refrigerated foods being transported across different countries and regions [5]. SARS-CoV-2 virions adhering to solid surfaces are reported to be stable, with a viability up to longer than 72 h (on plastic) [6]. Therefore, sensitive, fast, and low-cost SARS-CoV-2 detection is urgently needed for cold-chain foods.

SARS-CoV-2 is a spherical or pleomorphic enveloped particle containing single-stranded (positive-sense) RNA associated with a nucleoprotein within a capsid comprised of matrix protein [7]. The four main structural proteins encoded by COVID-19 are envelope protein (E protein), spike protein (S protein), membrane protein (M protein), and nucleocapsid protein (N protein) [8]. These four proteins are important for the viral infection of cells and replication and transcription [7,9]. Among them, S protein is a protruding protein widely distributed on the surface of the viral envelope that mainly mediates the fusion of the viral and host cell membranes [10]. It consists of two subunits, S1 and S2: S1 mainly contains the receptor binding domain (RBD), which is responsible for recognizing cellular receptors [11]. A recent study has shown that N-protein-based assays are more sensitive for detecting SARS-CoV-2 infection, while S-protein-based assays are more specific [12], and thus, S proteins are equally valuable as assay targets and can be used as biomarkers in antigen detection.

Currently, there are various detection methods for SARS-CoV-2, which are broadly classified into three categories: (1) Nucleic acid detection [13], including fluorescence quantitative PCR [14], microfluidic chip [15], Isothermal amplification technique [16], etc. Real-time fluorescence quantitative PCR (RT-qPCR) is the method for detecting viral RNA in samples from the upper respiratory tract, featuring high specificity, high sensitivity, and pre-infection detectability [13]. Since the outbreak of the epidemic, nucleic acid testing has been used as the “gold standard” for the diagnosis of SARS-CoV-2. (2) Serological testing [17] is a method for detecting the presence of SARS-CoV-2-specific antibodies (IgM/IgG) in a patient’s blood. (3) Antigen detection [18] is a method that utilizes specific monoclonal antibodies prepared against the antigenic proteins of SARS-CoV-2 to detect the intrinsic components of the virus, such as the N and S proteins of SARS-CoV-2, using immunofluidic chromatography [19], enzyme-linked immunosorbent assays [20], chemiluminescence [21], and other methods. One of them, the colloidal gold immunochromatographic assay [22], is widely utilized due to its simplicity, speed, and low cost for quick detection in food safety and public healthcare.

Lateral flow assays (LFAs) [19] are one type of diagnostic scheme that can provide rapid, point-of-care results. Aptamers are single-strand nucleic acid sequences that fold into secondary structures and have target binding affinities on par with antibodies [23]. As chemically synthesized agents, aptamers are also produced more affordably and reproducibly than antibodies [24], and also have other multifarious advantages such as good specificity, long-term stability during storage, ease to produce, liable modification, and meritable flexibility in different sensing formats, making them well suited for rapid virus detection. Currently, the application of the combination of aptamers and lateral flow assays to the development of target identification has yielded many results [25,26,27]. DNA aptamers against SARS-CoV-2 S protein have been successfully identified using an in vitro iterative library selection process called the systematic evolution of ligands by exponential enrichment (SELEX) [28,29].

The market for new crown assays, dominated by PCR, is indeed dominating at present. However, traditional nucleic acid detection methods, although accurate, are expensive in terms of instrumentation and require specialized equipment and personnel, which may be limiting in remote areas or emergency situations [30]. In contrast, aptamer test strip technology is simple to operate, has a short detection time, provides an intuitive visualization of results, and costs less than $1 per test to manufacture, which provides the advantages of accurate, portable, and efficient detection [27]. These features have allowed the test strip method to fill a market gap in certain specific scenarios and applications [31]. Especially in the early stages of the outbreak, due to the lack of supply of nucleic acid test kits and limitations in testing capacity, the test strip method played an important role as a complementary testing tool.

Secondly, aptamer test strip technology has a wide range of application areas [32], which can be used not only for clinical testing in medical institutions, but also in scenarios such as rapid screening in public places and personal self-testing, further expanding its market space. With the advancement of science and technology and in-depth research, test strip methods are being improved and optimized, and commercial applications will become more widespread. The investment and support from various parties such as governments, medical institutions, and enterprises for the new crown testing technology will also further boost the market. Innovations in technology offer more possibilities for the development of test strip methods in the new crown testing market [33].

In this work, the “universal” aptamer MSA52 was chosen as the detection aptamer, which showed generally high affinity for wild-type SARS-CoV-2 spike proteins, as well as Alpha, Beta, Gamma, Epsilon, Kappa, Delta, and Omicron proteins, with KD values ranging from 2 to 10 nM [29]. A lateral flow chromatography test strip based on nucleic acid aptamers using nanogold as a chromogenic agent was successfully developed, an aptamer engineering approach was used to select a capture aptamer with high binding affinity, and the application of the optimized conditions was successfully validated for the on-site screening of SARS-CoV-2 SP in cold-chain food packages, both visually and quantitatively.

## 2. Results

### 2.1. Characterization of AuNPs and Determination of Coupling Conditions

**Figure 1:**
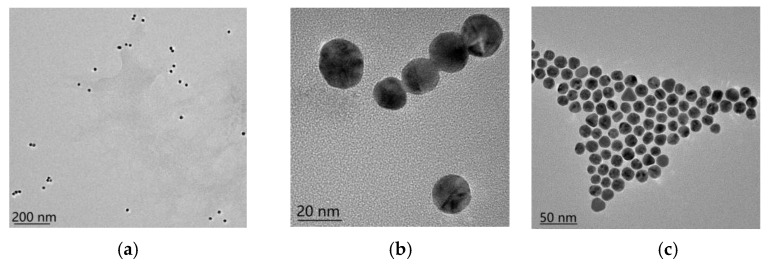
TEM test results. (**a**) AuNPs; (**b**) AuNPs; (**c**) AuNP–aptamer.

**Figure 2:**
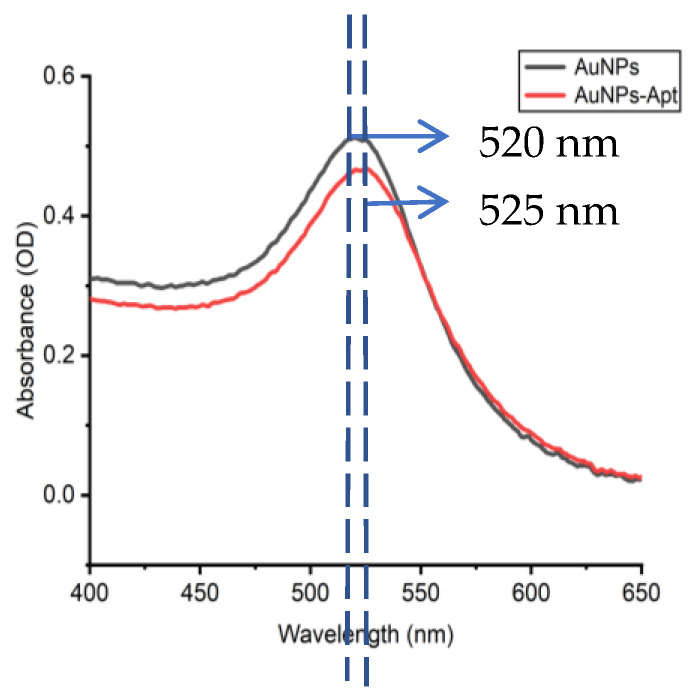
The UV absorption spectra of AuNPs and AuNP–aptamer conjugation.

The approximate binding state of gold nanoparticles (AuNPs) and nucleic acid aptamers can be determined by transmission electron microscopy. The results of the transmission electron microscopy characterization show (Figure 1a,b) that the AuNPs are spherical in shape, with a relatively homogeneous particle size (about 20 nm), and are well dispersed. From Figure 1c, it can be seen that the AuNPs after coupling maintain a regular arrangement of certain gaps between them, and no dispersion or condensation occurs. The comparison of the before and after pictures shows that the nucleic acid aptamer and the gold nanoparticles form a stable complex [34]. In addition, after the aptamer binds to AuNPs, the AuNPs become larger, and the absorption wavelength of the AuNP–aptamer is slightly shifted to the right under UV spectrophotometer characterization [35]. As shown in Figure 2, the UV absorption peak of bare AuNPs is at 520 nm, and that of the AuNP–aptamer is at 525 nm, with a rightward shift of 5 nm between the two.

**Figure 3:**
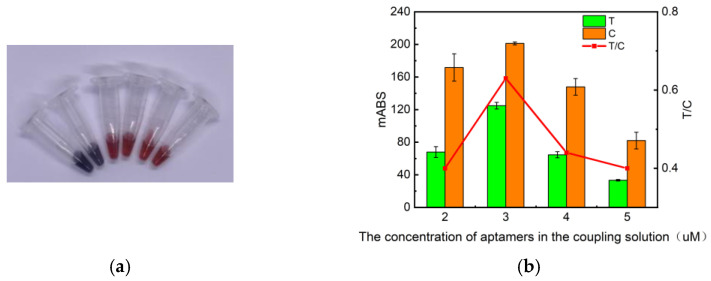
Effect of aptamer concentration on test strip results. (**a**) Physical diagram of coupling solution at different aptamer concentrations: from left to right, aptamer concentrations were 0.5 µM, 1 µM, 2 µM, 3 µM, 4 µM, and 5 µM. (**b**) Absorbance values of test strips measured by the immunochromatographic reader at different aptamer concentrations.

The determination of the aptamer concentration in the coupling solution is crucial to the experimental results. The aptamer added in the process of making the coupling solution was selected from the aptamer MSA52, with a high-affinity specific recognition ability for SARS-CoV-2 S protein, published by Zhang Zifeng et al. [29]. The coupled aptamer concentration gradients were set to 0.5 µM, 1 µM, 2 µM, 3 µM, 4 µM, and 5 µM, and the optimal amount of aptamer added was determined to be 3 µM by determining the OD_T_/OD_C_ through an immunochromatographic reader C10066-10 (Figure 3).

### 2.2. Optimization of Test Strip Conditions

#### 2.2.1. Optimization of SA Concentrations and Molar Ratios of SA to Biotin-DNAT

**Figure 4:**
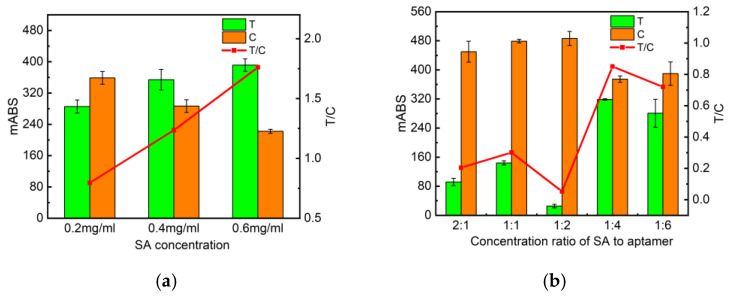

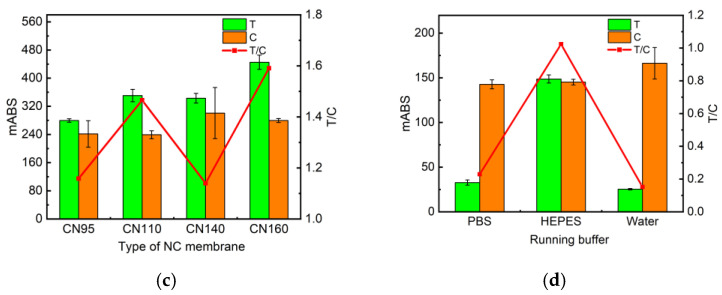
Optimization of test strip conditions. (**a**) Absorbance values of test strips measured by the immunochromatographic reader at different SA concentrations. (**b**) Absorbance values of test strips measured by the immunochromatographic reader at different aptamer concentrations at different ratios of SA to C- and T-line aptamers. (**c**) Absorbance values of test strips measured by the immunochromatographic reader with different NC membrane types. (**d**) Absorbance values of test strips measured by the immunochromatographic reader with different binding buffers.

The text line (T line) signal value is lower than that of the control line (C line) when the streptavidin (SA) concentrations of the T and C lines are equal from the experimental results, which may be because the complementary binding capacity of poly T-poly C of the C line is stronger than that of a certain segment of the DNA strand of the aptamer. So, we investigated the concentration of T-linear streptavidin (0.2 mg/mL, 0.4 mg/mL, 0.6 mg/mL), fixed the intensity of C-linear streptavidin at 0.2 mg/mL, and also examined the different molar ratios of SA to Biotin-DNA_T_ (2:1, 1:1, 1:2, 1:4, 1:6) for favorable probe fixation on nitrocellulose (NC) membranes. The higher the concentration of SA in the T line, the higher the signal level, and the best color development was achieved at an SA concentration of 0.4 mg/mL (Figure 4a) with a molar ratio of 1:4 (Figure 4b). Under these conditions, the absorbance of the test strips was satisfied and a comparable degree of color development of the T and C lines was reached.

#### 2.2.2. Optimization of NC Membranes

Here, we choose the four models of NC membranes: BSK95, BSK110, BSK140, and BSK160. As shown in the data of Figure 4c, the T/C values of BSK95 and BSK140 were closest to one and the absorbance of BSK140 was higher. As a result, BSK140 is the most suitable for this experiment.

#### 2.2.3. Optimization of Running Buffer

In this study, the signal values of the reactions under three buffers (PBS, HEPES, and water) were compared (Figure 4d), in which the HEPES buffer had the best color development response. It is basically consistent with the buffer composition used in the aptamer literature [29]. The specific compositions of the three buffers were set up as follows: (1) 50 mM of HEPES, 1% sucrose, 0.1% Tween-20, 2.5 mmol/L of CaCl_2_, 2.5 mmol/L of MgCl_2_, 6 mmol/L of KCl, 150 mmol/L of NaCl, and 1% BSA; (2) ultrapure water, 1% sucrose, 0.1% Tween-20, 2.5 mmol/L of CaCl_2_, 2.5 mmol/L of MgCl_2_, 6 mmol/L of KCl, 150 mmol/L of NaCl, and 1% BSA; and (3) 10 mM of PBS, 1% sucrose, 0.1% Tween-20, and 1% BSA.

### 2.3. An Aptamer Engineering Approach to Selecting T-Line Complementary Sequences

**Figure 5:**
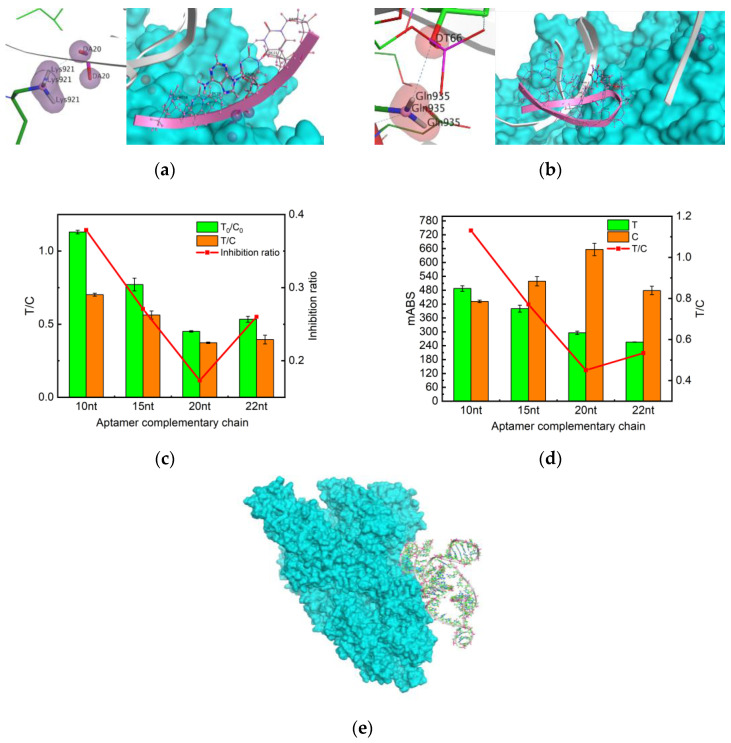
Molecular docking and aptamer-complementary chain element optimization. (**a**) DA20 docking site and its aptamer sequence. (**b**) DA66 docking site and its aptamer sequence. (**c**) Absorbance values of test strips measured by the immunochromatographic reader with different aptamer-complementary sequences with blank conditions. (**d**) Absorbance values of test strips measured by immunochromatography with different aptamer-complementary sequences with SP 500ppb conditions. (**e**) MOE molecular docking result.

From the analysis of aptamer and protein binding sites, DT80 and DT82 are on the extended sequence of the aptamer poly T [36] (Table 1). Therefore, the binding sites were identified as DA20 (Figure 5a) and DT66 (Figure 5b). The lower the binding energy of the site, the more stable the binding, which means that the binding effect is the best at DA20. After that, the aptamer sequence where DA20 is located was selected and extended by 10 nt, 15 nt, and 20 nt, and the complementary sequences of the resulting aptamers were MSA10, MSA15, and MSA20, in order.

Based on the MOE molecule docking results (Figure 5 and Table 1) and the selected aptamers as described in the original literature, it was finally decided to retain the four truncated aptamers, MSA52-10, MSA52-15, MSA52-20, and MSA52-22 (Table 2). In the presence of 500 ng/mL of SP, MSA52-10 manifested a high inhibition rate and good color rendering effect (Figure 5c,d).

### 2.4. Optimization of Detection Conditions

**Figure 6:**
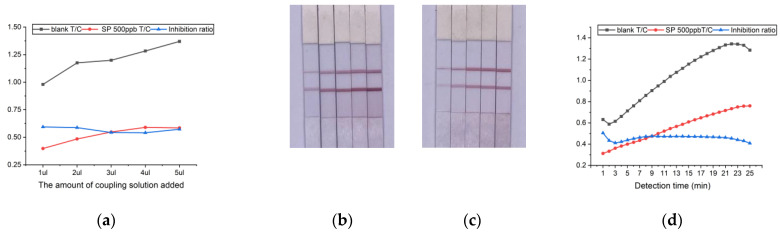
Optimization of detection conditions. (**a**) T/C and inhibition ratio with different amounts of coupling solution added. (**b**) Test strip experiments with different coupling solution spiking amounts under blank conditions, from left to right: 1 µL, 2 µL, 3 µL, 4 µL, and 5 µL. (**c**) Test strip experiments with different coupling solution spiking amounts under 500ppb SP conditions, from left to right: 1 µL, 2 µL, 3 µL, 4 µL, and 5 µL. (**d**) T/C and inhibition rate change over time.

Optimization of sample addition: The total spiked volume was set to 50 µL, and the spiked volume of nanogold–aptamer coupling (1 µL, 2 µL, 3 µL, 4 µL, 5 µL) was optimized. With the increment of the spiking volume, the color of the T and C lines progressively deepened, and OD_T_/OD_C_ gradually increased. Comparing the blank and spiking experiments (Figure 6), the color change was most pronounced at a spiking volume of 2 µL, and the highest inhibition rate was observed at this time.

The detection time was optimized: after the test strip was added to the sample, its OD_T_/OD_C_ value was read every 1 min from 1 min until the 25th min, and the results obtained are shown in Figure 6d. Calculation of the inhibition rate yielded an optimal detection time range of 8 min–15 min.

### 2.5. Quantitative Detection of SP by Test Strips

**Figure 7:**
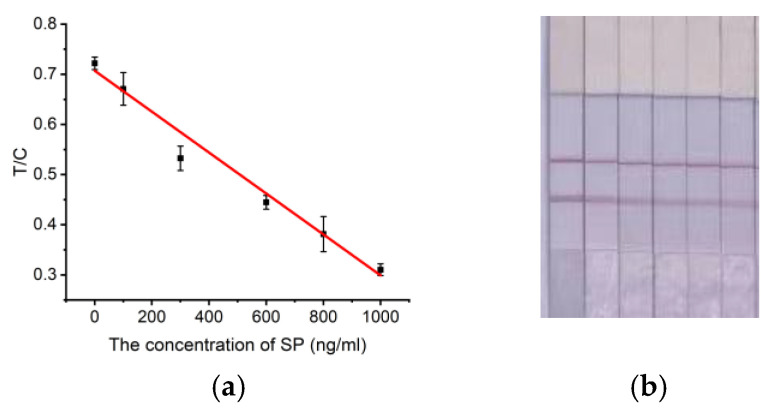
Quantitative detection of SP. (**a**) Standard curve. (**b**) Calibration curve and color development of test strips established at 15 min detection time, from left to right: 0 ng/mL, 100 ng/mL, 300 ng/mL, 600 ng/mL, 800 ng/mL, and 1000 ng/mL.

Test strips capable of visualizing and detecting SARS-CoV-2 S protein were constructed by incubating different concentrations of SP (0 ng/mL, 100 ng/mL, 300 ng/mL, 600 ng/mL, 800 ng/mL, and 1000 ng/mL) and AuNP–aptamer for 30 min and then performing spiking reactions for quantitative calibration (Figure 7). The higher the concentration of SP, the lighter the color of the area delineated by the T line, and its visual detection range is 0.1 µg/mL–1 µg/mL. The T/C ratio had a good linear connection with the SP concentration. y = −0.000408x + 0.70 (R^2^ = 0.97) was the linear equation at the detection time of 15 min, and the detection sensitivity of the initially constructed strip was calculated to be LOD = 91.2 ng/mL (≈0.68 nM) based on a 3 sigma/k scheme, where sigma refers to the standard deviation of the blank control and k refers to the slope of the linear equation. This suggests that the test strips we developed can measure SP at low levels and fulfill the requirements of the national SP detection standard. Although the sensitivity of the test strips prepared in this work is lower than other methods (Table 3), the efficiency and convenience of the test strip method in detecting and reading results on the basis of adequate visual detection are irreplaceable.

### 2.6. Specificity Test

**Figure 8:**
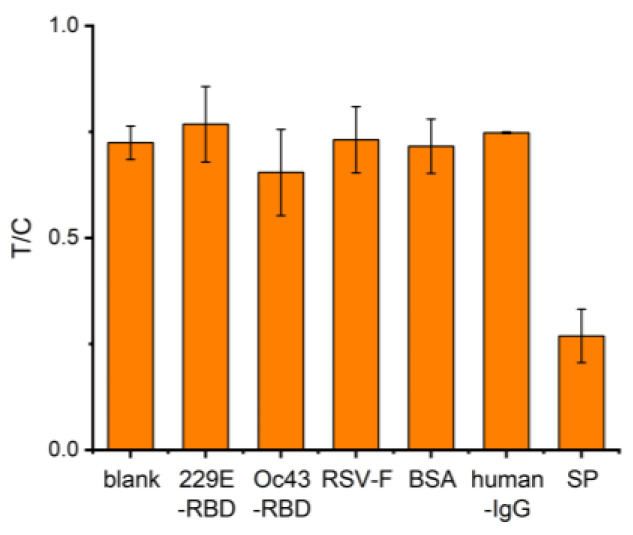
Results of a selectivity study of nucleic acid aptamer test strips for SP detection.

HCoV-229E-RBD protein, HCoV-OC43-RBD protein, RSV-F protein, human IgG protein, BSA, and SARS-CoV-2 SP were selected to perform the specificity contrast test under the concentration condition of 1000 ng/mL. As shown in Figure 8, the color of the T line of the S-protein group showed a significant decrease, and the rest of the absorbance had no significant change and was close to that of the blank control group, which indicated that the kit had good detection specificity.

### 2.7. Reproducibility Evaluation

In order to explore the inter-batch and intra-batch precision of the aptamer-based test strips prepared by this method, experimental verification was carried out. Three different batches of test strips prepared by this method were randomly selected, and three different concentrations of S protein (0.25 μg/mL, 0.5 μg/mL, 1 μg/mL) were used for sampling experiments. Each concentration in each batch was measured in parallel three times. The mean value ($\bar{X}$), standard deviation (SD), and coefficient of variation (CV = (SD/$\bar{X}$) × 100%) were calculated (Table 4). In general, a CV within 15% is considered to meet the precision requirements of the test strip. It was proven that the CV of the test strips prepared in the experiment was less than 15%, and the reproducibility was good.

### 2.8. Stability Assessment

**Figure 9:**
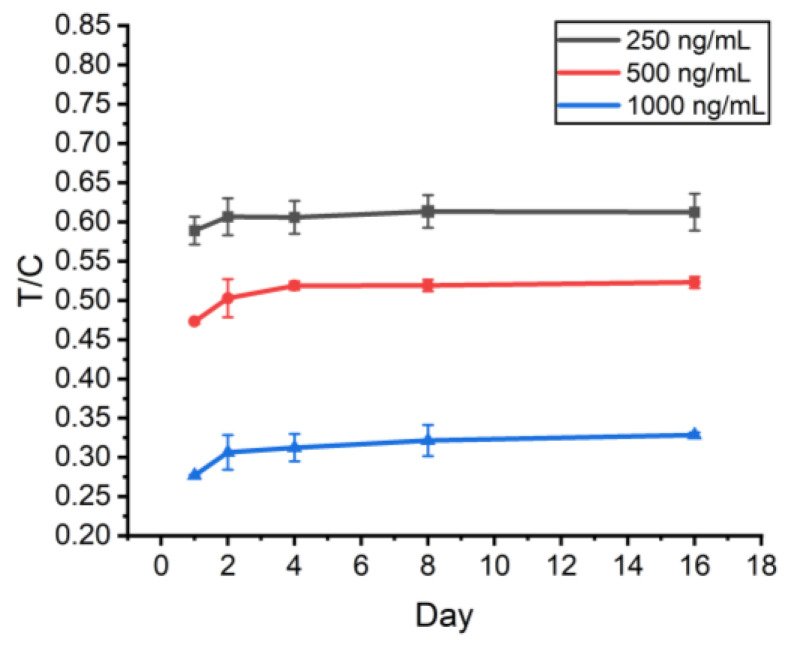
Stability evaluation result.

To evaluate the stability of the prepared test strips, the same batch of freshly prepared test strips was stored at room temperature (25 °C) and taken out on the 1st, 2nd, 4th, 8th and 16th days to detect different concentrations of S proteins (1000 ng/mL, 500 ng/mL, and 250 ng/mL). The results showed that the T/C values of the test strips increased slightly (within 15%) on the second day and then stabilized, and the test strips were still effective in detecting the three concentrations of S proteins (Figure 9), which indicates that the prepared test strips have a certain degree of stability at room temperature.

### 2.9. Recovery Assay

The above test strip was applied to the testing of frozen solutions on the surface of cold-chain food packaging with the addition of S-protein standard solution (0, 200, 400, 600, 800, 900, 1000 ng/mL), and the recoveries obtained ranged from 83.3% to 108.8% (Table 5) with relative standard deviations (RSDs) of 2.3% to 6.2%. The results showed that the lateral chromatographic test strips had high accuracy and reliability when applied to the detection of S protein in real samples.

## 3. Discussion

**Figure 10:**
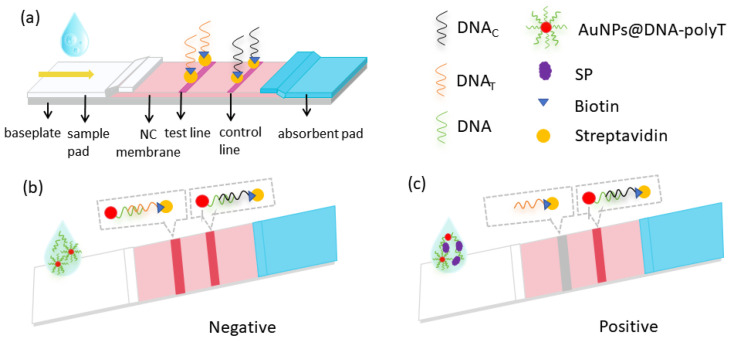
Schematic representation of SP detection via Apt-LFA. (**a**) Structure of LFA strip. (**b**) Negative result of Apt-LFA (without SP). (**c**) Positive result of Apt-LFA (with SP).

In this experiment, the principle of competition method was designed as shown in Figure 10, which is essentially a competitive interaction between the aptamer-complementary chain on the T line and the SP for AuNP–aptamer conjugates. The paper strip was formed by using AuNPs as a color developer, an aptamer coupled with AuNPs as the recognition element, and the aptamer-complementary chain as the capture probe. One end of the aptamer that binds to the S protein modifies sulfhydryl groups and couples AuNPs, and the other end adds 10 T bases (ploy T) to form a AuNP–aptamer-poly T complex. The sample solution flows along the NC membrane by capillary action. In the absence of SP in the sample solution, the aptamers in complexes will pair with the complementary pairing of the T line, while the DNA_C_ (poly A) immobilized in the C line binds complementarily to poly T, causing two red bands to appear; when SP exists in the sample, the AuNP–aptamers in the sample binds to the SP, thus weakening the binding force of the complementary sequences on the T line, and the color of the test line will become lighter. As the concentration of the SP is larger, the color of the T line becomes lighter until it disappears.

The effect of AuNP–aptamers is critical to the sensitivity of the assay. Due to electrostatic repulsion, colloidal gold produced by the reduction method of trisodium citrate is negatively charged by the encapsulation of trinitrate citrate anions and is distributed in an aqueous solution [42]. It is well known that, owing to the negatively charged colloidal, gold particles can be sheltered by Na^+^, which causes the colloidal gold particles to combine due to hydrophobic interactions and van der Waals forces. The aptamer should be covalently joined to the AuNPs through Au-S to create functionalized AuNPs. In terms of the three coupling methods (salt aging [35], freezing [43], and low pH), the solution coupled by the traditional salt-aging method is relatively time-consuming, but its coupled solution is the most stable and can be left for at least one month. In the coupling process, too little aptamer addition is not enough to protect the nanogold. Too much aptamer addition will result in too much free aptamer in the coupling solution, reducing the binding of the aptamer of the coupled AuNPs to the S protein, so that, even after the addition of the protein, there is still a free aptamer binding to the complementary chain. Therefore, the optimal amount of aptamer to be added was determined by the state of the coupling solution and the color development of the test strip.

In addition, another decisive factor is the choice of T-line aptamer-complementary chains. The aptamers screened from nucleic acid libraries generally have a length of ~80 bases, but the region that exerts target binding is only 10–15 nucleotides [44]. If the selected complementary chain is not its active site for protein binding, the complementary chain will still be complementary to the aptamer, even if the SP binds to the aptamer after adding the SP, resulting in a decrease in sensitivity, which is not conducive to the experiment. Therefore, MOE molecular docking was utilized to simulate effective binding sites and simplify the selection process of complementary sequences. On this basis, different complementary chain lengths were set to obtain the best reaction result. Finally, we decided to retain the four truncated aptamers, MSA52-10, MSA52-15, MSA52-20, and MSA52-22. Then, the blank and spiking experiments were carried out, respectively, in which the best color development and the highest inhibition rate were found to be for MSA52-10. This may be due to the fact that the base that is not at the effective site will still bind to the complementary chain, even if the aptamer and SP successfully bind. In this way, we can save costs and improve the sensitivity of the experiment.

Then, the conditions of the test strip were optimized. The coupling status of SA with biotin-modified probes not only impacts the amount of probe immobilization on NC membranes but also influences the binding efficiency of probes to nucleic acids [45]. The higher the SA concentration, the more aptamers are bound. However, too much SA will cover the aptamers, resulting in insufficient aptamers. Nitrocellulose membrane is the largest pad in the test strip, which is decisive for the sensitivity of the test strip [46]. Different NC membrane types have different pore sizes and chromatographic properties with different flow rates. In addition, the running buffer is vital for the spatial structure of the aptamer and directly contributes to the binding of the aptamer to the target [47]. Excessive addition of AuNP–aptamers will interfere with the experimental detection limit, and the addition of too little sample is not enough to develop the color. Finally, an effective detection time frame is the most important. We select the best reaction conditions by color rendering and absorbance.

In this study, a competitive transverse flow test strip with AuNPs as the marker was successfully established, which uses high-affinity aptamer-immobilized AuNPs for the detection of SARS-CoV-2 S proteins in cold-chain foods. y = −0.000408x + 0.70 (R^2^ = 0.97) was the linear equation, and the detection sensitivity was calculated to be LOD = 91.2 ng/mL (≈0.68 nM). Meanwhile, we examined the detection specificity of the aptamer test strip method and verified the analytical accuracy, stability, and practical application performance of this lateral chromatography test strip.

In this paper, we constructed an innovative assay kit to target multiple variants of SARS-CoV-2 SP using aptamer technology. To date, several studies have successfully detected SARS-CoV-2 SPs (Table 6). Compared with the LFA detection methods in other studies, this study obtained high detection sensitivity through molecular docking, the selection of complementary sequences of nucleic acid aptamers with high affinity, and the optimization of experimental conditions. And compared to other methods, although the detection limit of LFA detection methods is usually lower, the test strips have certain advantages in rapid detection and other aspects.

In the field of novel coronavirus detection, although PCR has long been considered the gold standard, the expensive specialized equipment required and the specialized operators limit its convenient application in many scenarios. In contrast, the LFA method can be used not only outside the laboratory, but even by patients themselves at home, and its long shelf life and lack of need for special storage conditions make it particularly suitable for use in developing countries, small outpatient care centers, and remote areas and battlefields. The test strips provide results in less than 20 min and cost less than USD 1 per test to manufacture in our hands. By enabling more frequent home testing, more readily available diagnostic tools (e.g., test strips) may reduce the burden of virus epidemics on the health care system.

Our research presents a test strip constructed using the spike protein as the detection target, which has certain potential advantages and application possibilities for virus detection in real clinical samples. The spike protein is the main surface protein of the SARS-CoV-2 virus. Compared to PCR detection and test strip methods targeting the nucleocapsid protein, the method developed in this research does not require virus lysis, thereby saving time and operational steps, and reducing the complexity of sample processing. In addition, the use of complementary strands as the test line not only reduces the cost but also ensures the reproducibility of the assay. Therefore, test strips based on the spike protein offer advantages such as rapidity, convenience, and non-invasiveness, facilitating rapid screening and diagnosis.

In conclusion, this study successfully applied the aptamer-based competitive method to SP detection, which not only improves the sensitivity and efficiency of new coronavirus detection, but also reduces the cost, provides an auxiliary that complements PCR detection, provides a new method and idea for virus detection, shows a convenient and fast application prospect in cold-chain food detection, and provides a new method for the development of alternative antibody detection reagents. After that, we can also try to develop multi-mode aptamer detection or multi-target detection, so as to develop a multi-functional test strip method for virus detection.

## 4. Materials and Methods

### 4.1. Reagents and Materials

The recombinant baculovirus-produced SARS-CoV-2 S protein (40589-V08B1) was obtained from Beijing Yiqiao Shenzhou Technology Co., Ltd. (Beijing, China). The sequence of aptamer binding to the SARS-CoV-2 S protein is 5′-SH-TTTTTTACGCCAAGGTGTCACTCCGTAGGGTTGGCTCCGGGCCTGGCGTCGGTCGCGAAGCATCTCCTTGGCGTTTTTTTTTT-3′, and the probe sequence of line C is biotin-AAAAAAAAAA. The other aptamer sequences are shown in Table 2. Aptamers and DNA probes were commercially synthesized and purified by Sangon Biotech Co., Ltd. (Shanghai, China). Streptavidin (SA) and bovine serum albumin (BSA) were ordered from Sigma-Aldrich (Saint Louis, MO, USA). Phosphate-buffered saline (PBS, PH 7.4) and 4-(2-hydroxyethyl)-1-piperazineethanesulfonic acid (HEPES) were purchased from Beijing Solarbio Company (Beijing, China). HAuCl_4_·4H_2_O, Proclin300, Tris(2-carboxyethyl)phosphine (TCEP), Tween-20, and sucrose were purchased from China Pharmaceutical Group Co., Ltd. (Beijing, China). Trisodium citrate and other reagents were purchased from Beijing Chemical Works. All inorganic chemicals and organic solvents were of at least analytical grade and buffer solutions were prepared with ultrapure water.

Plastic adhesive backing (6 × 30 cm), sample pads (8975), and absorbent pads (S270) were purchased from Hang Zhou Bulus Trading Co., Ltd. (Hangzhou, China), and the nitrocellulose (NC) membrane was obtained from Shenzhen Baisui Kang Industrial Co., Ltd. (Shenzhen, China). The NC membrane types and parameters are shown in Table 7. All plates were dispensed by an IsoFlow Dispenser (Imagine Technology, Wilmington, DE, USA) and then cut into strips for experiments using a programmable strip cutter (HGS201, AUTOKUN, Hangzhou, China). The signal values of the test strips were read by an immunochromatographic reader, the C10066-10 (Hamamatsu Corporation, Hamamatsu, Japan).

### 4.2. Preparation of Gold Nanoparticles

According to previous reports [54], AuNPs were prepared using a modified citric acid reduction method. Initially, 1 mL of a 1% HAuCl_4_ aqueous solution was added to 100 mL of ultrapure water in a 250 mL conical flask wrapped in tinfoil, stirred (300 rpm), and heated to boiling. Then, 2 mL of a 1% sodium citrate solution was quickly added while the rotational speed was adjusted to 350 rpm and maintained for 1 min. The solution continued to be heated for 14 min with stirring (300 rpm) until the color changed to burgundy, after which it was cooled to room temperature. The nanoparticles were stored at 4 °C away from light and were used at least 24 h after synthesis was completed.

### 4.3. Conjugation of Aptamer and Nanogolds

The conventional method for aptamer attachment on nanogold particles was typically performed by using a salt-aging process [35]: 50 μL of 5′-sulfide aptamer (3 μM) was activated by 1 μL of TCEP (10 mM) for 1 h at room temperature. During this time, 1 mL of the nanogold solution prepared above was concentrated 10-fold and the pH was reduced to 3.0. After mixing, the mixture was incubated for 2 h, and then 2 M NaCl was added to a final concentration of 75 mM over a 6 h period. Finally, it was stored at 4 °C for over 6 h until use.

### 4.4. Selection of T-Line Complementary Sequences

T-line complementary sequences were selected as per the original literature [29] and MOE molecular docking results. Secondary and tertiary structures of the aptamer were predicted by the DNA Fold Web server [55] and the 3dRNA/DNA Web server [56], and the structure of the S protein was obtained from PubChem [57]. The MOE was utilized to dock the aptamer to the SP to obtain the optimal binding site. All complementary chain sequences are shown in Table 2.

### 4.5. Pre-Treatment of Test Strips

The sample pad, NC membrane, absorbent pad, and PVC plastic adhesive backing are the four material elements that compose the aptamer-based lateral flow test strip [19]. To load the probe in the detection region, streptavidin (SA) was used as the connector between the probe and the membrane because of the adsorption of proteins by the nitrocellulose membrane. Utilizing biotin- and SA-specific binding properties, the probe, one end of which was modified with biotin and complementary to the AuNP–aptamer coupler, was immobilized onto the NC membrane. Fixed SA with a 1:4 molar ratio of aptamer, a T-line SA concentration of 0.4 mg/mL, and a C-line SA concentration of 0.4 mg/mL were incubated with biotin-DNA_T_ and biotin-DNA_C_, respectively, for 2 h to form SA-biotin-DNA complexes. The tubes were then washed three times for 30 min each with ultrafiltration centrifuge tubes (30 kD) to remove excess probe. The remaining solution was added to HEPES, and the final volume was the same as the original solution volume.

The sample pads need to be immersed in HEPES containing 1% BSA, 0.25% Tween-20, and 1% sucrose, dried at 37 °C, and stored in a cool, ventilated area to minimize non-specific adsorption between SA and AuNPs.

### 4.6. Assembly of Test Strips

The NC membrane, sample pad, and absorbent pad were sequentially laminated at 2 mm and pasted onto a plastic backing plate. Then, the T-line and C-line solutions were sprayed onto the NC membrane using an IsoFlow Dispenser and dried at 37 °C for 30 min. The T-line SA concentration was 0.4 mg/mL and the ratio of SA to biotinized aptamer (DNA_T_) was 1:4. The concentration of SA in line C was 0.2 mg/mL and the ratio of SA to biotinylated aptamer (DNA_C_) was 1:4. The test strip plate was cut into 4 cm strips using the HGS201 programmable strip cutter and stored in a self-sealing bag with desiccant.

### 4.7. Sample Test and Evaluation Methods

In this work, we used commercially available frozen solutions from frozen food packages as samples to verify the accuracy and reliability of aptamer-based lateral flow test strips. The ice on the surface of frozen food packages was melted at room temperature and mixed with different concentrations of SP to prepare sample solutions with different SP concentrations. Then, 2 μL of successfully coupled AuNP–aptamer, 28 μL of binding buffer, and 20 μL of sample solution were taken and incubated for half an hour and then added dropwise into the sample pad of the test strip. Ten minutes later, the absorbance of the T and C lines by immunochromatography was scanned and recorded with an immunochromatographic reader, the C10066-10.

The inhibition rate is introduced to evaluate the optimization results of various properties throughout this study and is calculated as follows [58]:

$$\mathrm{Inhibition}=\frac{\frac{T_{0}}{C_{0}}-\frac{T}{C}}{\frac{T_{0}}{C_{0}}}$$

T_0_ and C_0_ represent the absorbance of the T and C lines when there is no SP in the sample, while T and C represent the absorbance of the T and C lines when the sample has different S proteins. The absorbance ratio of T and C lines versus concentration was used for quantitative analysis and a standard calibration curve was plotted.

## Figures and Tables

**Table 1:** Aptamer and protein binding sites.

Number	Energy	Aptamer Binding Site	Protein Binding Site	Aptamer Sequence
1	−15.89	DA20	Lys921	DT18-DT22
2	−0.9	DT66	Gln935	DC65-DG71
3	−0.72	DT80	Lys285	DT77-DT84
4	−0.64	DT82	Lys278	DT77-DT84

**Table 2:** Truncated aptamer sequences and C-Line complementary sequence.

Name	Sequences (5′-3′)
MSA-10	Biotin-ACGCCAAGGA
MSA-15	Biotin-ACGCCAAGGAGATGC
MSA-20	Biotin-ACGCCAAGGAGATGCTTCGC
MSA-22	Biotin-CGCCAGGCCCGGAGCCAAACCC
Control-line DNA	Biotin-AAAAAAAAAA

**Table 3:** Detection of SARS-CoV-2 by strip method.

Target	Detection Range	LOD	Reference
S Protein	/	100 pM	[37]
N Protein	0.1–500 ng/mL	0.1–0.5 ng/mL	[38]
N gene	0.25–100 copies/mL	0.25 copy/mL	[39]
IgG	10 ng/mL–100 µg/mL	4 ng/mL	[40]
SARS-CoV-2 Virus	0–50 ng/mL	10 ng/mL	[41]
S Protein	100 ng/mL–1000 ng/mL	91.2 ng/mL	This work

**Table 4:** Precision evaluation results of the test strip.

Batch	SP Concentration (μg/mL)	$\bar{X}$ of T/C	SD	CV (%)
1	1	0.2717	0.0398	14.64
	0.5	0.4413	0.0565	12.81
	0.25	0.5780	0.0851	14.73
2	1	0.3519	0.0416	11.82
	0.5	0.5032	0.0669	13.30
	0.25	0.6602	0.0873	13.23
3	1	0.3122	0.0396	12.70
	0.5	0.4710	0.0696	14.78
	0.25	0.6535	0.0967	14.80
Intra-batch	1	0.3119	0.0403	13.05
	0.5	0.4718	0.0643	13.63
	0.25	0.6305	0.0897	14.25

**Table 5:** Results of spiked recovery experiments of new crown S protein in cold-chain food pouch samples (*n* = 3).

Sample	Concentration of SP (ng/mL)	Detection Result	Test Strip Concentration (ng/mL)	Recovery Rate (%)	RSD (%)
Cold-chain food packaging bags—tap water rinsing (1 mL)	0	Negative	Undetected	Undetected	Undetected
	200	Positive	166.7	83.3	2.6
	400	Positive	435.2	108.8	2.3
	600	Positive	571.7	95.2	5.8
	800	Positive	752.4	94.1	6.2
	900	Positive	860.2	95.6	4.6
	1000	Positive	968.1	96.8	3.0

**Table 6:** Detection of SARS-CoV-2 S protein by biosensors.

Method	Target	Detection Range	LOD	Reference
E-AB	S Protein (S1)	0.001–1000 fg/mL	1 ag/mL	[48]
SPR	S Protein (S1)	1–100 nM	0.26 nM	[49]
Fluorescence (FL)	S Protein	10 fg/mL–10 ng/mL	7.8 fg/mL	[50]
PEC	S Protein	75 fg/mL–150 pg/mL	1.22 fg/mL	[51]
LFA	S Protein (RBD) S Protein (S1)	62.5–4000 ng/mL 250–4000 ng/mL	62.5 ng/mL 250 ng/mL	[52]
LSPR	S Protein (RBD)	2.03–9420 pM	0.83 pM	[53]

**Table 7:** The NC membrane types and parameters.

Name	Speed (s/4 cm)	Diameter (µm)
BSK95	100 ± 20	12–15
BSK110	120 ± 30	8–12
BSK140	140 ± 30	5–8
BSK160	160 ± 30	4–6

## Data Availability

All data can be easily obtained and linked in the respective sections.
